# Electronic sanitary database: a new potential tool to identify occult chronic liver disease in general population

**DOI:** 10.1007/s11739-023-03507-1

**Published:** 2024-01-16

**Authors:** Silvia Cagnin, Andrea Martini, Daniele Donato, Paolo Angeli, Patrizia Pontisso

**Affiliations:** 1 Unit of Internal Medicine and Hepatology (UIMH), Department of Medicine (DIMED), Padova Teaching Hospital, Padua, Italy; 2 Medical Head Office, Padova Teaching Hospital, Padua, Italy

**Keywords:** Chronic liver disease, Cirrhosis, Epidemiology, Early diagnosis

## Abstract

Chronic liver disease (CLD) is a leading global cause of mortality, morbidity, and healthcare resource utilization. However, the burden of CLD is underestimated because the course of the disease is often asymptomatic until clinical decompensation and the development of life-threatening complications. In this study, we assessed the use of available blood tests from electronic medical records for identifying individuals with undiagnosed CLD in the general population. We analyzed a total of 202,529 blood tests obtained from 99,848 adults recorded in the Electronic Health Records of the Padova Teaching Hospital. Transaminases levels > 1.5 times the normal value indicated occult CLD, while platelet counts < 120,000/μL identified occult cirrhosis. We characterized patients using Italian Medical Exemptions (IME), excluding oncologic cases. Overt and occult cirrhosis prevalence was 1% and 4.18%, respectively, while overt and occult CLD affected 2.85% and 4.61% of the population. The epidemiology of patients with overt and occult cirrhosis was similar but significantly different from that of the controls. Among subjects aged 60–70 years, working disability was twofold higher in those with occult cirrhosis compared to those with overt cirrhosis. Occult CLD and cirrhosis had higher prevalence rates than diagnosed cases in the general population. Electronic medical record data may serve as a valuable tool for CLD identification, potentially reducing cirrhosis development and clinical decompensation. This, in turn, may lead to a decrease in the economic impact on the healthcare system.

**Supplementary Information:**

The online version contains supplementary material available at 10.1007/s11739-023-03507-1.

## Introduction

Chronic liver disease (CLD) is a leading cause of mortality, morbidity, and health care resource utilization globally [1, 2]*.* Currently, liver cirrhosis provokes 1.16 million deaths, with liver cancer accounting for 788,000 of those; these conditions rank among the top 20 causes of death worldwide, causing 3.5% of all global mortality [3, 4]. Nevertheless, the burden of CLD is underestimated [5], as the course of the disease is often asymptomatic until clinical decompensation, with the development of life-threatening complications (e.g., gastrointestinal bleeding, ascites, and jaundice), when the only cure is liver transplantation. However, due to the organ shortage, less than 10% of global organ transplantation needs are met [4]. The economic impact of liver cirrhosis is high [6] and has outpaced other common chronic diseases, such as chronic heart failure (CHF) and chronic obstructive pulmonary disease (COPD), involving a younger population who suffers from a low quality of life [7]. It has been estimated that the peak age of mortality occurs in the late 40 s and early 50 s, with liver disease being one of the leading causes of years of working life lost in Europe, second only to ischemic heart disease [8].

Chronic hepatitis B virus (HBV), hepatitis C virus (HCV), alcohol-related liver disease (ALD), and non-alcoholic fatty liver disease (NAFLD) are the most common causes of CLD and cirrhosis. Recently, the introduction of an effective treatment for HCV infection and vaccination campaigns for HBV, alongside an increased prevalence of obesity and alcohol abuse in general population, are reshaping the epidemiology of CLD [5, 8]. Chronic hepatitis B and C are a major public health challenge, and together with tuberculosis, malaria and HIV infection are in the focus of the World Health Organization (WHO) who wanted to achieve their elimination by 2030 [9, 10]. Though, the HCV detection and elimination require financial resources that are even a challenge for developed countries. It should be noted that estimates indicate that fewer than a half of HCV-infected individuals have been identified and that only a minority of known carriers are treated, due to the difficulties of population screening and the cost of testing and treatment [11]. The new available drugs against hepatitis C have been allowed to successfully treat 700,000 patients for HCV in 2015, who represent only 1% of the total infected population (71 million people globally) [4]. HBV vaccination has reduced the prevalence of this infection in children, leaving unaltered the burden of chronic HBV infection in older generations [8].

The other two main causes of CLD are due to modifiable drivers (alcohol consumption and NAFLD/NASH), and the earlier the damage is known the greater the potential for behavioral changes and other treatment to be effective and limit or even halt the progression of the disease [12]. Europe has the highest alcohol consumption rates globally, leading to over 50% of end-stage liver disease (ESLD) cases and making it the most common reason for liver transplantation [8].

The prevalence of NAFLD/NASH is increasing worldwide proportionately to the increase in epidemic of obesity and type 2 diabetes mellitus [1, 13], already representing the most common liver disease in the world [8]. In the European Union, NAFLD is estimated to affect 20–30% of the population, with around 3% of patients progressing to NASH [14]. Predictions suggest that, unless significant measures are taken, NAFLD is likely to emerge as the leading cause of end-stage liver disease (ESLD) in Europe as well [8]. Despite the increasing knowledge in the pathogenesis and treatment of alcoholic and non-alcoholic chronic liver disease, the majority of patients are still diagnosed at an advanced stage of disease [15, 16], resulting in a huge economic burden [17, 18].

These premises highlight the need to identify liver disease early through prevention campaigns and screening, focusing on preventing disease progression rather than managing end-stage liver disease [8]. As emphasized in the Baveno VII consensus [19], a critical aspect for patients with CLD is the prevention of first decompensation, which can be achieved by treating the primary etiological factor of the liver disease. Appropriate therapies targeting potential precipitating events (e.g., non-selective beta-blockers in patients with esophageal varices, primary and secondary antibiotic prophylaxis in patients with encephalopathy) could prevent disease progression and improve prognosis [20–22]. Furthermore, for patients with overt cirrhosis, biannual ultrasound exploration of the liver in expert hands is mandatory for HCC surveillance, leading to early diagnosis and potentially curative treatment [23].

To this purpose, validated risk tools and prediction models are under investigation to help general practitioners to identify asymptomatic patients who are at risk of developing liver cirrhosis [24] or patients with undiagnosed silent and compensated cirrhosis. Indeed, it has been recently estimated that a large proportion of patients with chronic liver disease followed by the primary care service are still undiagnosed, and liver blood test results could help to recognize patients who need preventative health intervention or need to be referred to secondary care [25]. Then the widespread use of electronic health records could help primary care providers to recognize patients at higher risk for CLD [26].

The aim of this study was to assess the use of specific blood tests to subsequently validate an ACG (Adjusted Clinical Groups) system from electronic medical records, to identify subjects with unrecognized chronic liver disease in the general population.

## Materials and methods

### Epidemiological analysis

For the epidemiological analysis, we considered 202,529 blood tests obtained from 99,848 adults in the outpatient setting, recorded in the Electronic Health Record of Padova Teaching Hospital between January 1 and December 31, 2016, in the context of the Regional Project “Application of a decision support system for the early diagnosis of liver disease” (PRIHTA-2014-00000460). The data were anonymously extracted from the database by the Information Technology personnel, and they included epidemiological data (age and sex), blood test results, and Italian Medical Exemptions (IME) codes.

### Markers of overt liver disease and other conditions

**Fig. 1:**
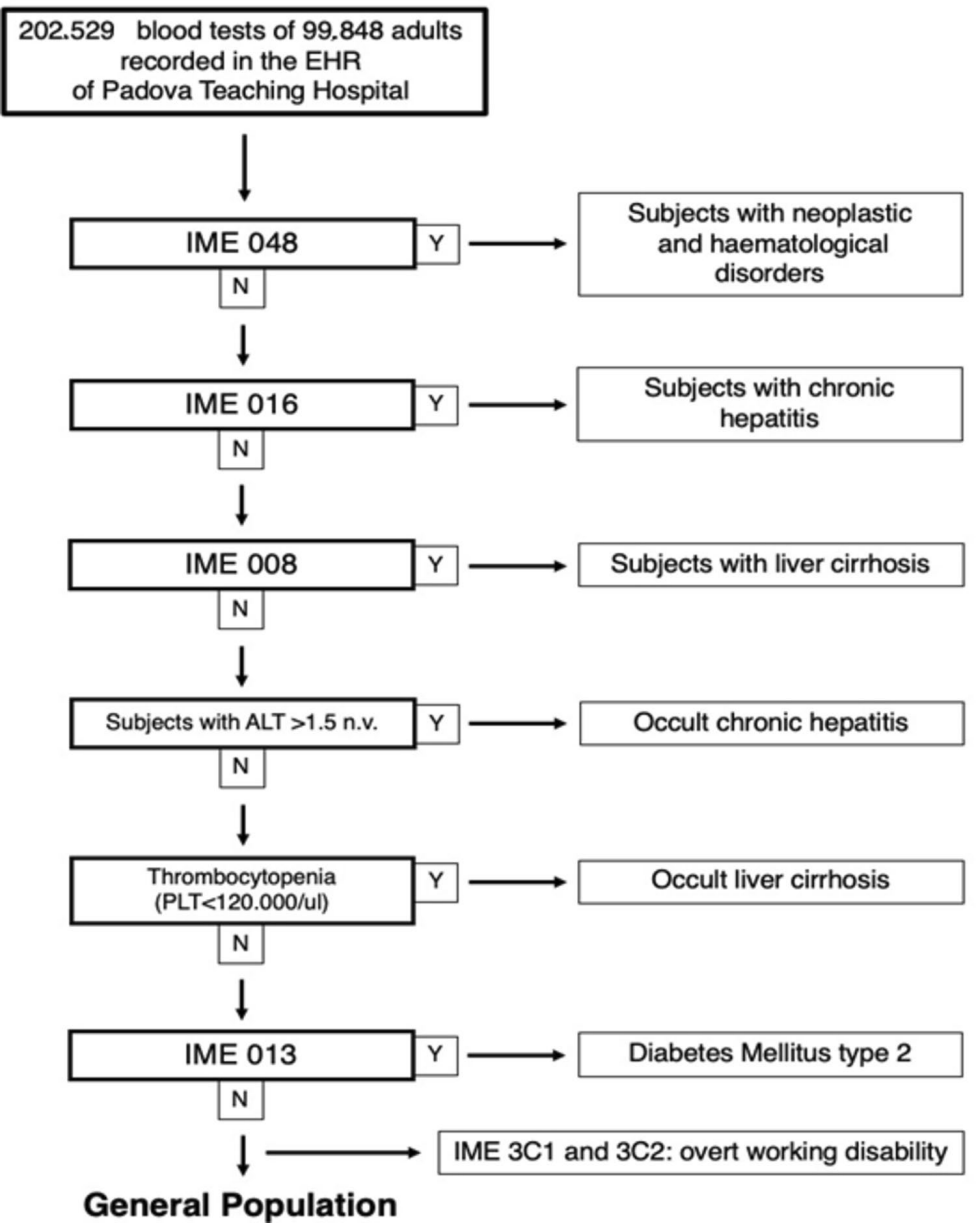
The flowchart used to identify subjects with occult chronic liver disease and occult liver cirrhosis. *EHR* electronic health record, *IME* Italian Medical Exemption, *Y* yes, *N* no

Patients with overt CLD and cirrhosis were defined as subjects with an already known diagnosis of CLD and cirrhosis, identified by the Italian Medical Exemption (IME) codes of chronic hepatitis and liver cirrhosis (Supplementary Table 1). The presence of diabetes and overt working disability was also determined by IME codes recorded in the electronic sanitary database. Individuals without any of these characteristics were used as controls. We excluded, from the analysis, patients with neoplastic and hematological disorders that had been previously identified through IME (Fig. 1). In addition, patients with discordant values were also excluded to ensure alterations were not due to other medical conditions.

### Surrogate markers for occult liver disease

Subjects with occult CLD were defined as subjects without IME of chronic hepatitis, liver cirrhosis, hematological or neoplastic disorders, with values of ALT > 1.5 n.v. on at least two occasions with an interval of at least 1 month. Subjects with occult cirrhosis were defined as subjects without IME of liver cirrhosis, chronic hepatitis, hematological or neoplastic disorders, with thrombocytopenia (platelet count < 120,000/mL) [25, 27, 28] on at least two occasions with an interval of at least 1 month. Also in this case, patients with discordant values were excluded.

### Statistical analysis

The prevalence of overt and occult chronic liver disease, cirrhosis and its comorbidities was calculated by dividing the number of affected individuals by the total number of subjects tested in the overall population. Normally distributed continuous variables (e.g., age) were reported as means with standard deviations and compared with Student’s *t* test or one-way ANOVA test for multiple comparisons when appropriate. Categorical variables were reported as proportions and compared using the Chi-square test with continuity correction.

Data analyses were conducted using the SPSS PC statistical package (SPSS, Inc., Chicago, IL). All reported *p *values are two-tailed.

## Results

The prevalence of IME for CLD and liver cirrhosis was found to be 2.85% and 1%, respectively. Alterations in transaminases (ALT), considered markers of occult CLD, were observed in 4.61% of the subjects, while thrombocytopenia, a surrogate indicator of occult cirrhosis, was detected in 4.18% of the subjects (Table 1).

**Table 1:** Prevalence of liver disease and epidemiological characteristics of patients included in the study

	Overt CLD	Occult CLD	Overt cirrhosis	Occult cirrhosis	Overall population
Prevalence	2.85%	3.77%	1%	4.18%	
Age (years)	56 ± 14*	68 ± 18	62 ± 11**	64 ± 13	70 ± 16***
M/F ratio	1.08^c^	1.23^d^	1.71^a^	1.88^b^	0.60

Overt cirrhosis and overt and overt chronic liver disease (CLD) were classified by IME, Italian Medical Exemption; occult cirrhosis was defined as platelet count < 120,000 m/l; occult chronic liver disease (CLD) was defined as transaminase (ALT) above 1.5 normal values in at least two occasions

*Overt CLD vs. occult CLD: *p* < 0.0001 (Student’s *t* test); **overt cirrhosis vs. occult cirrhosis: *p* < 0.0001(Student’s *t* test); ***one-way ANOVA comparison among all columns: *p* < 0.0001

^a^overt cirrhosis vs. overall population: *p* < 0.0001

^b^occult cirrhosis vs. overall population: *p* < 0.0001

^c^overt CLD vs. overall population: *p* < 0.0001

^d^occult CLD vs. overall population: *p* < 0.0001

The epidemiological characteristics of patients with overt or occult CLD and cirrhosis in the study population are summarized in Table 1. Patients with overt or occult liver cirrhosis were similar in age and younger than the controls included in the study. Interestingly, individuals with occult CLD were slightly older than those with overt CLD (66 years old vs. 56 years old), possibly due to the asymptomatic nature of their underlying condition. In terms of gender distribution, patients with overt and occult CLD and cirrhosis were more frequently male, consistent with literature data*.*

**Fig. 2:**
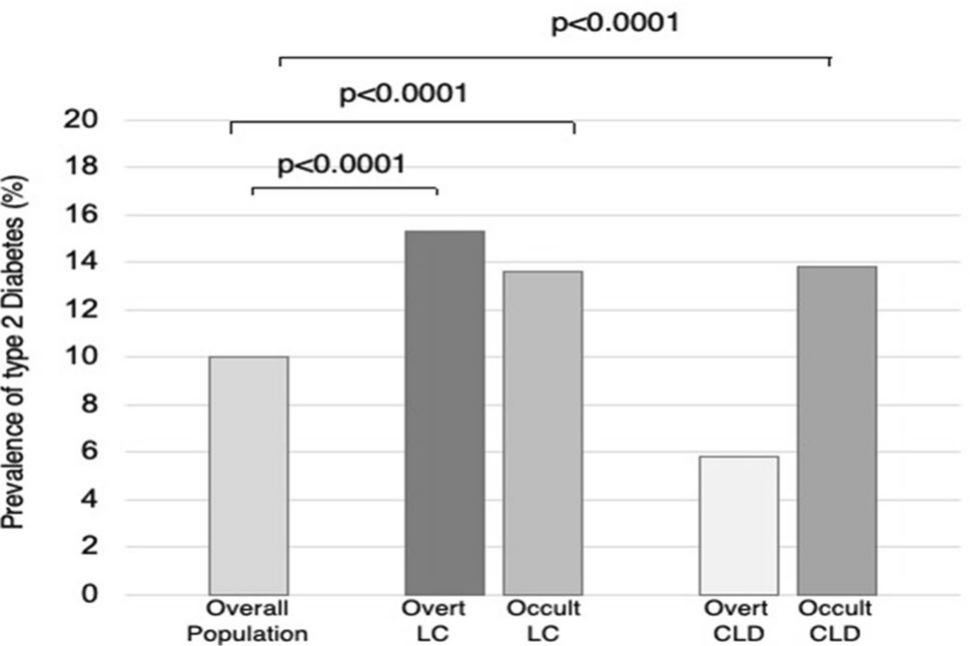
Prevalence of diabetes mellitus in different populations. Prevalence of diabetes mellitus type II in patients with overt and occult chronic liver disease and cirrhosis and in overall population. *LC* liver cirrhosis, *CLD* chronic liver disease

Regarding diabetes mellitus type II, the main comorbidity associated with NAFLD and NASH, patients with overt cirrhosis showed a higher prevalence of diabetes (15.3%) than the general population, and similar patterns were observed in patients with occult cirrhosis (13.6%) (Fig. 2). Within the CLD population, patients with overt CLD had a lower prevalence of diabetes, while it was noteworthy that patients with occult CLD had a prevalence closer to that of patients with cirrhosis (13.8%) (Fig. 2), suggesting that unrecognized NASH can frequently occur in this population. This prevalence is consistent with literature data [29, 30], although more recent studies suggest the percentage may be even higher [31, 32].

**Fig. 3:**
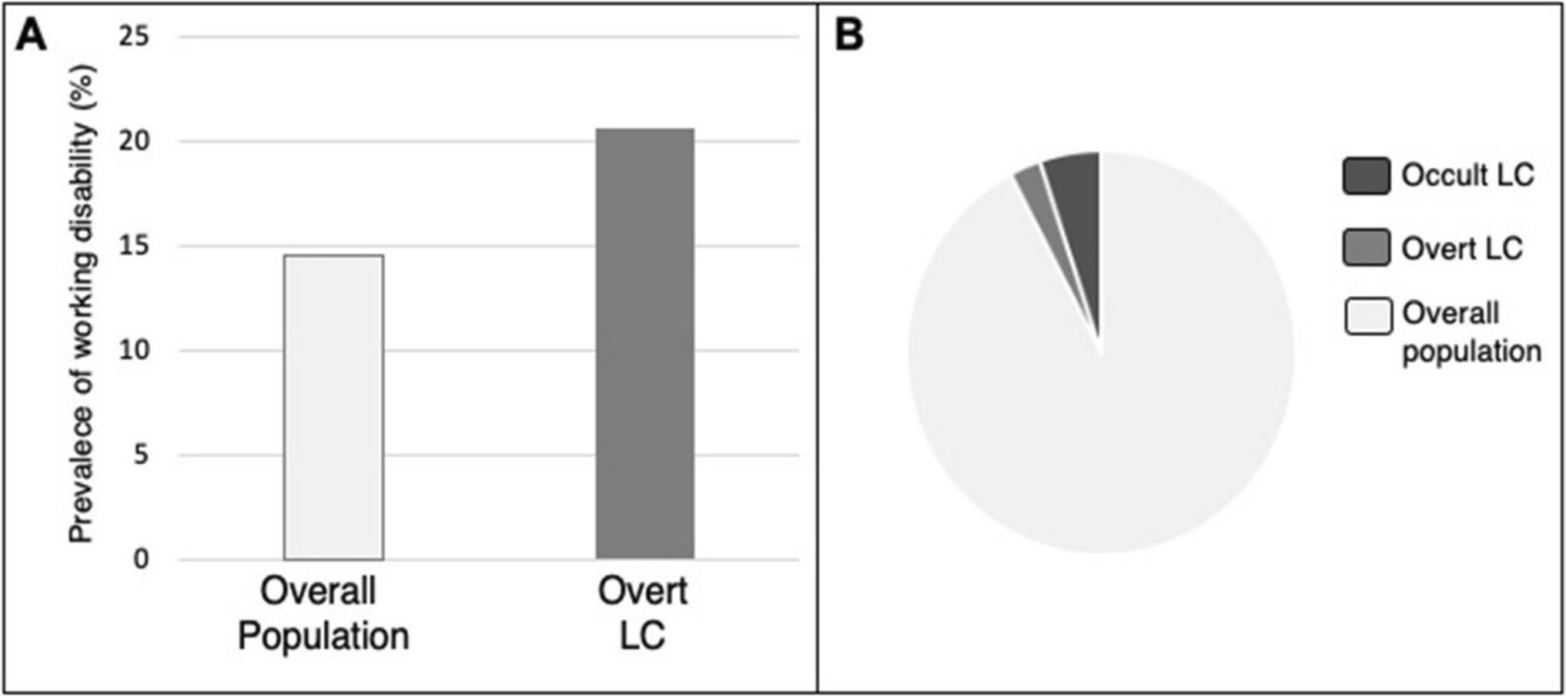
Working disability and liver cirrhosis. Prevalence of overt working disability in patients with liver cirrhosis and control population (2a). Distribution of patients with overt and occult liver cirrhosis among patients with IME for working disability (2b). *LC* liver cirrhosis

Patients with overt cirrhosis had a higher prevalence of overt working disability compared to the control population (20.6 vs. 14.5%, Fig. 3a), highlighting the social impact of this health condition. To delve deeper into this aspect, an analysis among patients between 60 and 70 years old was conducted, revealing that the prevalence of overt working disability in this selected population was 4.9%. An unexpected relevant finding was that, among this subgroup, patients with overt cirrhosis had a prevalence of 2.9%, while the figures rose to 5.13% in patients with occult liver cirrhosis (Fig. 3b).

## Discussion and conclusion

In this study, our goal was to screen for unrecognized liver disease in a population undergoing blood chemistry tests for other medical reasons in the outpatient setting, using data from the Electronic Health Record of Padova Teaching Hospital. Primary care typically manages the majority of patients at high risk of chronic liver disease [33], so screening through medical records could be a useful tool for identifying these patients.

Currently, liver diseases are screened for through blood abnormalities. In our study, we used elevated transaminases as a surrogate indicator for CLD and a low platelet count as a surrogate indicator for cirrhosis. Transaminases can fall within the normal range in many individuals with CLD and cirrhosis, making these patients difficult to detect [8]. However, other studies highlighted their high sensitivity and specificity in identifying CLD [25, 27, 28]. A low platelet count was considered an indicator of occult cirrhosis after excluding other possible causes, such as hematological disorders. This indicator was chosen because it is frequently measured in routine blood tests and is closely associated with cirrhosis due to portal hypertension and splenomegaly.

In the analyzed population, both occult cirrhosis and CLD (4.18% and 4.61%) had a prevalence four and two times higher, respectively, than overt disease. The population with occult CLD tended to be older compared to those with overt CLD, possibly due to the fact that these individuals often have an undiagnosed metabolic syndrome, which is typically asymptomatic. This hypothesis is also supported by the high prevalence of diabetes, which was significantly higher than that of the control population, as is expected due to the increasing prevalence of NAFLD [5, 34]. The reliability of our approach was further supported by the finding that in occult liver disease, especially occult cirrhosis, males were predominant, as expected from existing literature.

Liver diseases can be treated, and their progression can be slowed down when detected early. The 2022 EASL-Lancet commission estimates that prevention and early detection could save 300,000 lives across Europe each year [8]. Screening for CLD could also be cost-effective and can result in better social outcome. Gines et al. have highlighted how non-invasive screening strategies for risk stratification result in quality-adjusted life-years (QALYs) falling below the threshold set for therapies in most countries. The importance of non-invasive screening strategies lies in the opportunity cost of reallocating the same budget from the treatment of CLD and end-stage liver disease to the prevention of cirrhosis and its decompensation [33]. As liver diseases affect a young population, preventing liver disease could not only increase the quality of life, but also reduce the years of working life loss. Early identification of liver disease could also be a useful approach for patients with metabolic syndrome, for which multidisciplinary teams of specialists are needed for a comprehensive management.

The type of screening we have analyzed has shown the ability to identify occult liver disease with the advantages of being easy to perform and cost-effective, without adding further expenses to the healthcare system, and it is not time-consuming for the patient. Furthermore, general practitioners could easily refer patients for further testing when laboratory results suggest occult liver disease.

It should be noted that this study has some limitations. First, this is an opportunistic screening, looking for additional disease in a population already presenting medical issues, so it cannot be said that the screened population is supposedly healthy. In addition, to confirm the presence of occult liver disease, imaging techniques should be performed, but this was out of the purpose of the present study and will be the goal of further studies. Another issue is that this type of screening could leave out disadvantaged socioeconomic groups, where unhealthy alcohol and food consumption are most prevalent [8], as individuals belonging to these socioeconomic groups tend to undergo fewer screening tests and be less invested in their own health. The target population included only people who underwent blood tests in a single center in Padova, so further studies are needed to achieve results more representative of the general population.

In conclusion, a large proportion of the general population shows biochemical parameters of chronic hepatitis and of liver cirrhosis, with a prevalence that is significantly higher than that of diagnosed patients. Data from electronic medical records of the general population could become a useful tool for the identification of subjects with occult liver disease, thus limiting cirrhosis development and its clinical decompensation, with an expected reduction of the economic impact on the healthcare system.

### Supplementary Information

Below is the link to the electronic supplementary material.

Supplementary file1 (PDF 15 KB)

## Data Availability

Data used in the study are available upon request.
