# Supplementary material for: Electronic sanitary database: a new potential tool to identify occult chronic liver disease in general population
Source: Intern Emerg Med. 2024 Jan 16;19(3):641–7. doi: 10.1007/s11739-023-03507-1 (PMC11039494; doi:10.1007/s11739-023-03507-1)
Supplement: Supplementary file 1 — Supplementary file1 (PDF 15 KB) [file 11739_2023_3507_MOESM1_ESM.pdf]

JOURNAL: Internal and Emergency Medicine

ELECTRONIC SANITARY DATABASE: A NEW POTENTIAL TOOL TO IDENTIFY OCCULT CHRONIC LIVER DISEASE IN GENERAL POPULATION

[1]Cagnin S, [1]Martini A, [2]Donato D, [1]Angeli P, [1]Pontisso P.

[1] Unit of Internal Medicine and Hepatology (UIMH), Department of Medicine (DIMED), University-Teaching Hospital of Padova, Padua, Italy

[2]Medical Head Office, Padova Teaching Hospital, Padova, Italy

Corresponding Author: Prof. Patrizia Pontisso

Dept. of Medicine, University of Padova

Via Giustiniani 2, 35123 Padova , Italy

e-mail: patrizia@unipd.it

SUPPLEMENTARY MATERIAL

Supplementary Table 1. Italian Medical Exemption codes used in the study.

**Diseases and conditions**	**IME**
Liver cirrhosis	008
Chronic hepatitis	016
Neoplastic disorders	048
Diabetes Mellitus type 2	013
Working disability	3C1 and 3C2

IME, Italian medical exemption
